# Veterinarians’ Knowledge, Attitudes and Practices Associated with Bovine Viral Diarrhoea Virus Control and Prevention in South-East Australia

**DOI:** 10.3390/ani10091630

**Published:** 2020-09-11

**Authors:** Claire McMorrow, Allan J. Gunn, Shahid Khalfan, Marta Hernandez-Jover, Victoria J. Brookes

**Affiliations:** 1 School of Animal and Veterinary Sciences, Charles Sturt University, Wagga Wagga, NSW 2650, Australia; cv.mcmorrow@gmail.com (C.M.); algunn@csu.edu.au (A.J.G.); shahid.khalfan@gmail.com (S.K.); mhernandez-jover@csu.edu.au (M.H.-J.); 2 Graham Centre for Agricultural Innovation (NSW Department of Primary Industries and Charles Sturt University), Wagga Wagga, NSW 2650, Australia

**Keywords:** bovine viral diarrhoea virus, Australia, control, persistently infected, welfare

## Abstract

**Simple Summary:**

Bovine Viral Diarrhoea Virus is a disease of cattle that causes production losses. Despite the virus being widespread across Australia, there are no government or industry-led programs to mitigate the impacts or eliminate Bovine Viral Diarrhoea Virus. Veterinarians were surveyed about their knowledge, attitudes and recommended practices regarding Bovine Viral Diarrhoea Virus and its control. We found that veterinarians’ knowledge of Bovine Viral Diarrhoea Virus in their region is limited, and their attitudes and recommendations for controlling the virus do not always align with those of producers. For example, veterinarians are concerned about the welfare and potential for disease spread associated with control measures involving persistently infected cattle, including a previously undocumented practice in which producers administer blood from persistently infected cattle into naïve cattle as a form of vaccination. This study highlights that a greater understanding of producers’ and veterinarians’ values is needed before Bovine Viral Diarrhoea Virus control could be implemented at a regional or country level.

**Abstract:**

In Australia, the responsibility and associated costs for the control and prevention of Bovine Viral Diarrhoea Virus (BVDV) rest solely with producers. Veterinarians provide producers with farm-specific options for BVDV management and support BVDV control and elimination in their region. We surveyed veterinarians to determine their knowledge, attitudes and practices (KAP) associated with BVDV control in south-east Australia. We found that veterinarians’ recommendations do not always align with producers’ control measures. Veterinarians were uncertain about BVDV prevalence and the proportion of producers using BVDV control measures in their regions. Veterinarians generally promoted biosecurity and vaccination, and were concerned about the welfare and additional disease risks associated with persistently infected (PI) cattle. Veterinarians highlighted concerns about disease risks associated with a previously undocumented practice in which producers collect blood from PI cattle to administer to BVDV naïve cattle; termed “vampire vaccination” in this study. A greater understanding of the burden, impact and economics of BVDV is needed to align veterinarians’ and producers’ KAP to improve BVDV management on farms, and more appreciation of veterinarians’ and producers’ values is needed before BVDV control could be implemented at a regional or country level.

## 1. Introduction

Bovine Viral Diarrhoea Virus (BVDV/Pestivirus) is a globally endemic, infectious disease of cattle that causes immunosuppression and substantial production losses, including reproductive losses, such as reduced conception rates, embryonic death, congenital deformities, abortions and stillbirths [1,2]. When infected with the non-cytopathic strain, the disease is often mild and subclinical. During transient viraemia (4–7 days), infected animals might suffer diarrhoea, pyrexia, nasal discharge and immunosuppression [3,4]. However, infection of dams with the non-cytopathic strain in early to mid-gestation, can result in the production of a persistently infected (PI) calf [5]. These calves are often chronically ill-thrifty, with reduced growth and survival rates due to prolonged immunosuppression and other infection-associated effects [1]. Infection of a PI with the cytopathic strain of BVDV can lead to mucosal disease with animals presenting with anorexia, diarrhoea, nasal discharge and ulcerative lesions [3].

The Australian cattle industry has been estimated to incur financial losses of AUD 114 million annually due to the impacts of BVDV infection [6]. Despite the substantial financial consequences of BVDV, there are no formally structured and freely accessible BVDV control programs coordinated by producer groups, or state and federal governments in Australia. This is most likely because BVDV losses are indirect, making them difficult to observe at an individual farm level. There is also limited information about regional BVDV prevalence in Australia because the available literature is limited and is often not herd-type specific or climate specific [7,8,9,10]. Additionally, many producers are unaware of the BVDV status of cattle on their property [11]. Diagnosis of BVDV infection is necessary to establish and then implement control practices, and it is especially important to detect PI cattle [12]. Such cattle are the primary source of BVDV transmission, due to life-long, abundant viral shedding [2,13,14]. The virus persists in the calf’s tissues following birth, and naïve cattle directly exposed to its bodily secretions can become infected. Some producers introduce PI cattle to their herd to maintain herd-immunity to BVDV [2,13]. Such cattle might carry and transmit other pathogens, which can lead to further disease in an immunologically naïve herd; therefore, losses might exceed those expected solely from BVDV [15]. These additional losses are also increased by the immunosuppressive nature of transient BVDV infection [16].

The implementation of control and management practices at farm level is not common in Australia [11], even though producers have indicated a willingness to implement management practices, particularly if they are economically beneficial [17]. Such practices can include herd surveillance (for example, to eliminate PI calves), increasing herd immunity, and improved biosecurity. Biosecurity is defined as actions that are implemented to prevent pathogens from entering a herd and minimise the spread of pathogens within a herd [18,19,20]. Increased herd immunity can be achieved by vaccination or exposure of naïve cattle to PI cattle (often termed “deliberate exposure” when used as a control method) [21,22,23]. Pestigard^®^, an inactivated vaccine, is the only commercially available vaccine within Australia [24]. It is effective against strains of subgenotypes BVDV-1a and BVDV-1c [22]; however, in Australia, BVDV-2is believed to be absent, with 97% of isolates within Australia confirmed as BVDV-1c [16,25].

Climatic differences, as well as herd size, also influence herd management practices which can influence BVDV risk; therefore, these must also be accounted for when implementing BVDV control [7,26]. Although the responsibility for control rests solely with producers, veterinarians are important in their capacity as advisers and clinicians on livestock properties and could provide farm-specific information to producers to reduce BVDV associated losses, as well as support regional or country-wide control programs.

The success of control and elimination of BVDV in other countries (for example, Ireland, France, Norway and Scotland) has highlighted different approaches to country-wide elimination. Differing perceptions of the importance of the impacts of BVDV mean that policies and attitudes relating to BVDV elimination vary between countries [9,10,27,28,29]. Countries in which BVDV has been eliminated introduced compulsory control programs based on the identification and subsequent removal of PI animals (the ‘test and eliminate’ method), leading to a reduction in both PI prevalence and herd-level incidence [23,30]. This was successful in Switzerland, in which steps initially aimed to improve the education of the industry and producers, followed by antigen testing of the entire cattle population and subsequent elimination of PIs [29,31].

Sweden and Norway implemented movement restrictions on BVDV antibody positive animals, while countries such as the United Kingdom and Ireland (countries in which BVDV control programs have yet to lead to elimination) use vaccination as well as the test and eliminate method [30].

Challenges exist in relation to control and elimination programs; for example, who should pay, and whether they should be voluntary or compulsory [27]. These challenges highlight a need for country or region-specific assessment of current knowledge, attitudes and practices of industry stakeholders, as well as bio-economic assessments to evaluate the benefits of eradication [9]. Such assessments in Sweden and Norway suggest the benefits of BVDV include elimination, improved animal welfare and production, enhanced industry reputation, and advantages in overseas markets [10,30,32]. However, there are substantial differences in the structure of cattle industries, including herd management and government regulations, as well as differing climates between Australia and the European countries that have successfully implemented BVDV control.

To assess the feasibility of improving the control of BVDV in Australia, we need to understand the current levels of knowledge, as well as attitudes and practices associated with BVDV management [33]. Veterinarians play a key role in disease control. However, to provide adequate advice to producers on BVDV management, veterinarians require knowledge of the prevalence, disease risks, efficacy of control measures and the associated costs and benefits. Veterinarians’ values also need to align with those of producers. Therefore, the objective of this study was to describe veterinarians’ knowledge, attitudes and practices (KAP) associated with BVDV control, relative to the practices and perceived attitudes of producers in their regions in south-east Australia. We aimed to demonstrate the range of KAPs of veterinarians (rather than determine majority opinions)—with a particular focus on the use of PI calves and deliberate exposure—to assess alignment of veterinarians’ and producers’ KAPs regarding BVDV control.

## 2. Materials and Methods

A survey of cattle veterinarians currently practising in south-east Australia was conducted. The study region was defined as the temperate climate zone in south-east Australia according to the Köppen climate classification of Australia [34]. The study was approved by the Charles Sturt University Human Research Ethics Committee (H19149).

### 2.1. Questionnaire Design and Implementation

The questionnaire design was informed by individual discussions with six industry and veterinary experts, including cattle veterinarians and past and current beef and dairy producers. Researchers used a semi-structured interview approach for these discussions, to gain background information to guide the questionnaire design. Topics included in these discussions were recommendations for BVDV control and practices, including those associated with PI calves such as deliberate exposure, and whether producers tested for BVDV and other diseases.

The questionnaire included 35 questions about veterinarians’ knowledge, attitudes and recommended practices and their perceptions of producers’ attitudes and practices associated with BVDV control. Initially, demographic information was collected, followed by the type of properties visited (beef, dairy farms or both), and the frequency of visits. Questions then differentiated BVDV management on beef and dairy properties, and on BVDV infected and uninfected properties. Positive herds were those with a known presence of recently BVDV seropositive cattle (due to natural exposure) or presence of PI cattle, and negative herds had a perceived negative status. Finally, questions focused on deliberate exposure using PI calves, the biosecurity risks that this might pose, and the health and welfare of PI calves. The questionnaire was piloted on the previously mentioned group of experts to improve clarity and refine questions.

### 2.2. Distribution

The questionnaire was distributed in electronic form via an online provider (Survey Monkey^®^, Australia, https://www.surveymonkey.com, accessed 9 September 2020). Cattle veterinarians were invited to participate by email if they were currently registered with the Australian Veterinary Association (Australian Cattle Veterinarians Special Interest Group) or the Australian and New Zealand College of Veterinary Scientists (Cattle Chapter) organisations. The questionnaire was available to these groups from 24 July 2019 to 28 August 2019. Although each distributor invited participation once, members of both organisations may have received two invitations.

### 2.3. Data Analysis

Data from the questionnaire was downloaded into Excel (Microsoft Corporation, version 16.16.13 (190811)) and analysed in the statistical platform, R [35]. All responses from practitioners in local government areas (LGAs) that extended over the temperate climate zone were included in analysis. Quantitative data were summarised using descriptive statistics and plots of distributions. Statistical calculations were completed with different denominators because the number of responses for each question varied; responses were not compulsory, and some questions were specific to participants’ type of work. Data from quantitative responses which included minimum, maximum and most likely estimates were combined as PERT distributions. Statistical significance between distributions was tested using Kruskal-Wallis tests. Qualitative responses were reviewed and discussed by all authors to summarise participants’ opinions.

## 3. Results

### 3.1. Demographics

**Figure 1:**
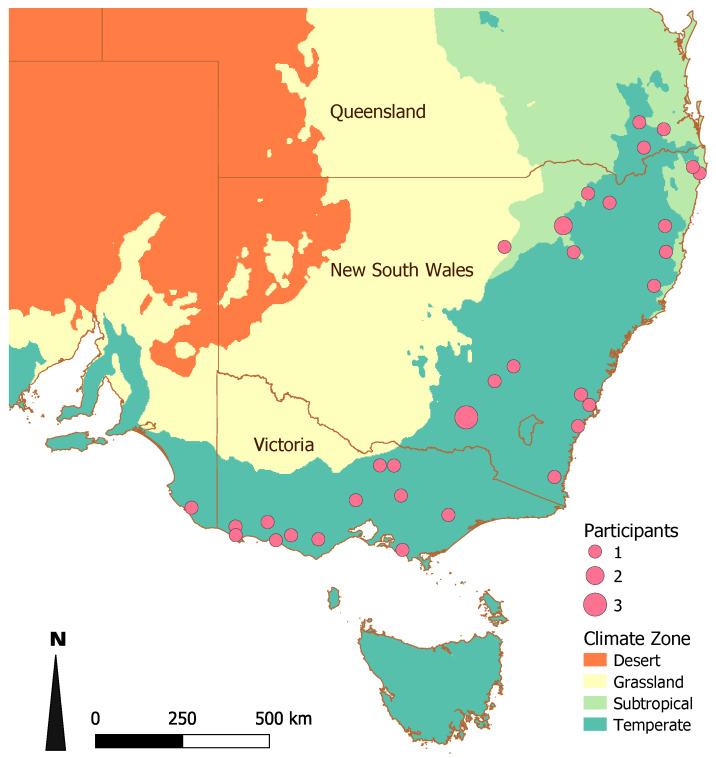
Location of participating veterinarians in a survey of veterinarians’ knowledge, attitudes and practices associated with bovine viral diarrhoea virus management on farms in the temperate zone of south-east Australia in 2019. Each dot is located on the centroid of the participant’s local government area (LGA); all LGSs extended into the temperate climate zone.

The questionnaire was completed by 48 veterinarians, of whom eight were excluded because they were not currently practising cattle veterinarians (*n* = 3), or were either not within the climate zone for this study or did not state their workplace postcode (*n* = 5). Of the remaining veterinarians (*n* = 40) who described their type of practice (*n* = 35), 48% worked with beef producers (*n* = 19), 15% worked with dairy producers (*n* = 6), and 25% worked with both beef and dairy producers (*n* = 10). Veterinarians most commonly visited one to five cattle producers each week (43%, *n* = 17; Supplementary Materials Figure S1). Most of the veterinarians were male (70%, *n* = 28), but in the youngest age group (<30 years old) there were more females than males (71% female, *n* = 5; data are presented in Supplementary Materials Figure S2). Most veterinarians were from New South Wales (56%, *n* = 24) and Victoria (28%, *n* = 12; Figure 1). The response rate for the questionnaire was not available because we did not have access to the databases of the distributing organisations.

### 3.2. Estimates of Herd-Level Prevalence of BVDV

**Figure 2:**
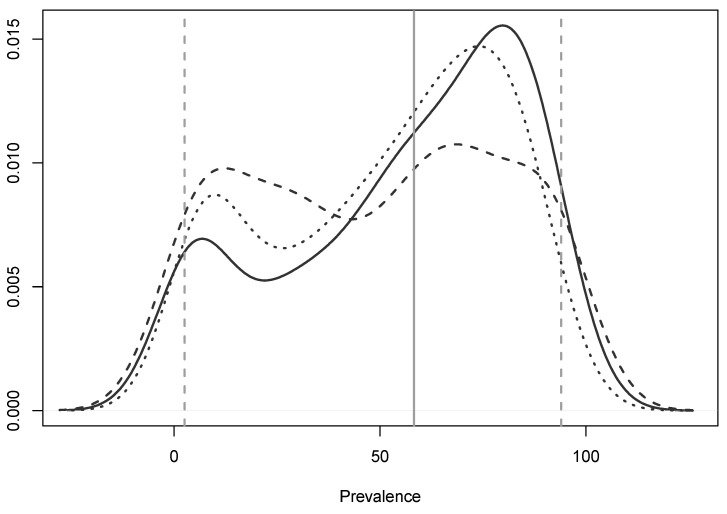
Density plot of the distribution of the estimated herd-level prevalence of bovine viral diarrhoea virus (BVDV) in beef breeder (black dotted line), beef rearer (black solid line) and dairy properties (dashed line) in a survey of veterinarians’ knowledge, attitudes and practices associated with BVDV management on properties in the temperate zone of south-east Australia in 2019. Vertical lines: grey solid = median combined between-property prevalence; grey dashed = 95% range of between-property prevalence. The extents of the plot <0 and >100 should not be interpreted; line extension is due to the smoothing process in constructing density plots.

There was no significant difference in the veterinarians’ estimated herd-level prevalence of BVDV on beef breeder (median 57%, 95% range 4–91%) and beef rearer (median 64%, 95% range 2–94%) properties throughout the veterinarians’ areas (*p* = 0.50) (Figure 2). For all property types, veterinarians’ estimates of herd-level prevalence in their areas appeared to follow a bimodal distribution of either low (approximately 5–10%), or, more commonly, high (approximately 80–90%) prevalence. The combined breeder and rearer beef properties’ median estimated herd-level prevalence was 61% (95% range 3–93%). Veterinarians estimated a median herd-level prevalence of 53% (95% range 2–95%) on dairy properties. Although the median estimated herd-level prevalence of BVDV was greater for beef properties than dairy properties, this was not a statistically significant difference (*p* = 0.50).

**Figure 3:**
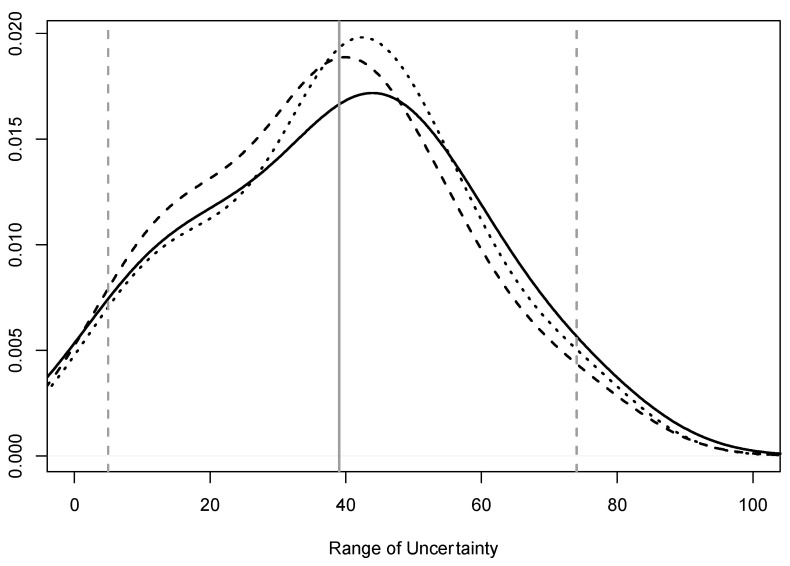
Density plot of the range of uncertainty about between-farm prevalence of bovine viral diarrhoea virus (BVDV) in beef breeder (dotted line), beef rearer (solid line) and dairy properties (dashed line) in a survey of veterinarians’ knowledge, attitudes and practices associated with BVDV management on properties in the temperate zone of south-east Australia in 2019. Vertical lines: grey solid = median combined between-property prevalence; grey dashed = 95% range of between-property prevalence. The extents of the plot <0 and >100 should not be interpreted; line extension is due to the smoothing process in constructing density plots.

Most vets were moderately uncertain about the herd-level prevalence in their area of work (median 39%; 95% range 5–74%; Figure 3). The main tests reported to be used by veterinarians were Agarose Gel Immunodiffusion (AGID; *n* = 15), PCR (*n* = 17) and ELISA (*n* = 27) (Supplementary Materials Figure S3).

### 3.3. Control and Prevention Practices

Veterinarians estimated that the proportion of BVDV positive properties on which control measures were implemented was 32%, although this varied widely (95% range 1–92%). Veterinarians similarly estimated that the proportion of BVDV negative properties on which preventive measures were implemented was 26% (95% range 2–87%).

**Figure 4:**
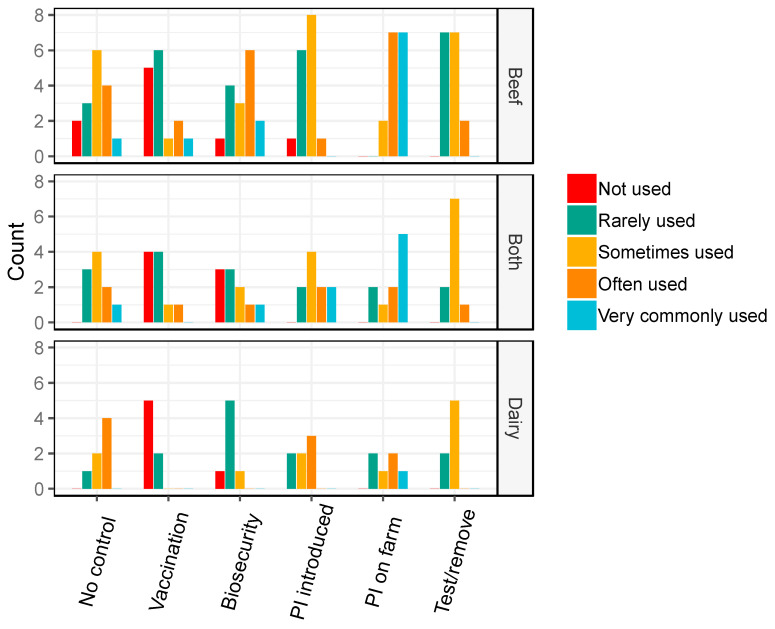
Barplots of the frequency of management practices used on bovine viral diarrhoea virus (BVDV) positive properties reported by veterinarians, in a survey of veterinarians’ knowledge, attitudes and practices associated with BVDV management on properties in the temperate climate zone of south-east Australia in 2019. PI = persistently infected cattle. Both = veterinarians working with both beef and dairy properties.

**Figure 5:**
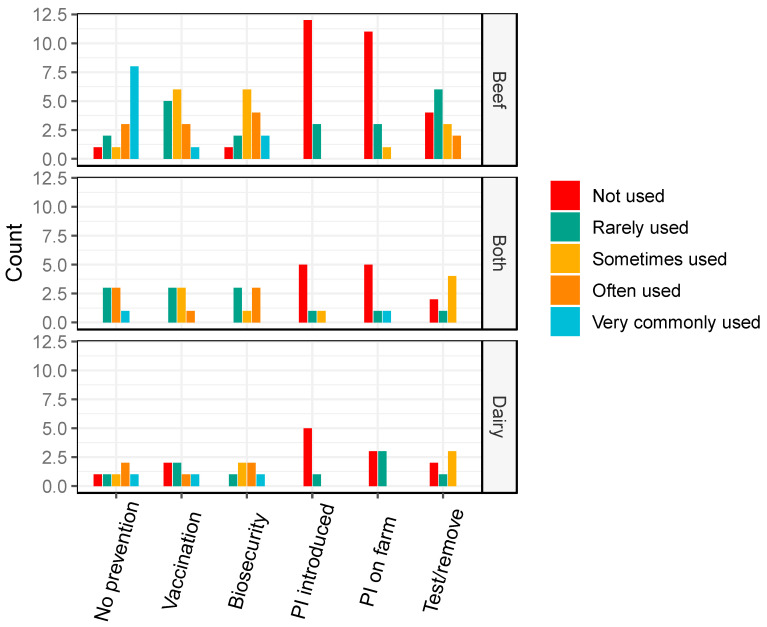
Barplots of the frequency of management practices used on bovine viral diarrhoea virus (BVDV) negative properties reported by veterinarians, in a survey of veterinarians’ knowledge, attitudes and practices associated with BVDV management on farms in the temperate zone of south-east Australia in 2019. PI = persistently infected cattle.

Vaccination was reported as more commonly used on BVDV positive properties than BVDV negative properties, and “no prevention” was relatively commonly reported on BVDV negative properties (Figure 4). Biosecurity on BVDV positive properties was estimated as “rarely” used on dairy properties and “often” used on beef properties (Figure 4), and “sometimes” used on BVDV negative properties (beef and dairy; Figure 5).

When asked how biosecurity was achieved on BVDV positive properties (an open question), veterinarians reported that producers used a range of measures. These included testing and quarantine of introduced stock, avoiding introducing pregnant stock, segregation of introduced and pregnant stock and improved perimeter fence control. While quarantine of acute infections was suggested to occur, there was little suggested in regard to determining PI status.

In response to an open question describing how producers employ preventive biosecurity practices (on perceived negative properties), veterinarians stated that it was achieved through bioexclusion, which included maintaining closed herds, testing of any introduced stock, and fencing. Veterinarians reported that maintaining a closed herd was more frequently associated with BVDV negative than BVDV positive properties. However, selective purchasing was sometimes practised and included only purchasing tested cattle (e.g., bulls) and obtaining a vaccination history before purchase. While determining the efficacy of prevention strategies was not an objective of this study, some veterinarians suggested that producers who had an awareness of the disease consequences tended to employ adequate biosecurity measures.

Veterinarians also reported that the use of PIs that were sourced on farm were “often” and “very commonly” used on BVDV positive properties, especially beef properties (Figure 4). In contrast, the introduction of PIs was reported less frequently overall on all positive property types. As expected, the use of PI cattle, vaccination and “test and remove” strategies were generally rarely used on BVDV negative properties (Figure 5).

When asked what measures veterinarians would recommend for BVDV control, veterinarians recommended vaccination (*n* = 20), and combined biosecurity and vaccination (*n* = 9). For prevention of BVDV, veterinarians most commonly recommended vaccination (*n* = 16) and biosecurity (*n* = 7). Deliberate exposure (*n* = 2), and the opposite strategy, the identification and removal of PIs (*n* = 7), were less often recommended overall.

### 3.4. Deliberate Exposure

Overall, veterinarians did not support the use of deliberate exposure to control BVDV (supporters = 2; non-supporters = 11), but some veterinarians said support of deliberate exposure was dependent on the producer’s circumstances (*n* = 16). Most veterinarians (*n* = 21) were willing to work with producers who used deliberate exposure, regardless of their own personal support for the practice, although two were not willing to do so because of welfare concerns.

There were mixed responses to open questions about the use of deliberate exposure that gave more insights about veterinarians’ attitudes and practices relating to deliberate exposure. Although most veterinarians recommended biosecurity and vaccination, some veterinarians suggested the use of deliberate exposure instead of vaccination. Others said that vaccination was an alternative when deliberate exposure was not feasible (*n* = 2) and deliberate exposure was termed “auto vaccination” by some veterinarians (*n* = 3). Some veterinarians also suggested that advice from veterinarians about BVDV control was influenced by producers’ willingness to control the disease. For example, by working with producers using deliberate exposure, veterinarians can “educate and harness their [producers’] enthusiasm into the right direction”. Two veterinarians specifically recommended education to improve producers’ understanding of BVDV to achieve effective control on BVDV positive properties.

When asked about PI acquisition, veterinarians reported a range of methods. Some producers actively sought PIs from within their own or other properties. Some inadvertently introduced PIs from newly acquired pregnant stock. Veterinarians also reported that young stock were more likely to be deliberately exposed to PI cattle than older stock (Supplementary Materials Figure S4). Veterinarians perceived that a driver for using PI cattle by producers was economic pressure (*n* = 12) and thought that producers perceived deliberate exposure as a cost-effective control method (*n* = 11) compared to vaccination. Some veterinarians (*n* = 3) also suggested that the longer lasting immunity generated from natural infection was a reason that producers used deliberate exposure. Some veterinarians also stated that producers used deliberate exposure due to “ignorance of the costs of disease” and “poor veterinary advice”.

### 3.5. Infectious Pathogen Risks Associated with PI Cattle

Most veterinarians (*n* = 26) believed that the introduction of PI cattle posed disease risks other than BVDV to a herd. Pathogens mentioned by veterinarians were grouped by researchers according to body system affected during clinical disease (Table 1). The combined (beef and dairy) median prevalence of enteric diseases within herds and the prevalence in PI cattle was estimated as 30% and 40%, respectively (distributions not significantly different; Kruskal-Wallis Χ^2^ = 11.65, df = 9, *p* = 0.23; Supplementary Materials Figure S5).

The prevalence of respiratory diseases was estimated with combined medians of 27.5% within herds and 50% in PIs (distributions not significantly different; Kruskal-Wallis Χ^2^ = 12.11, df = 9, *p* = 0.21). Systemic diseases were reported as a potential risk by 18 veterinarians. Combined estimated prevalence both within herds and in PIs was low (median 17.5% and 3.5%, respectively, distributions not significantly different; Kruskal-Wallis Χ^2^ = 3, df = 3, *p* = 0.39). While only reported by three veterinarians, “other” diseases (infectious kerato-conjunctivitis [pinkeye] and lice) on beef properties were estimated to have a high within herd and PI prevalence (median 80% and 70%, respectively).

Veterinarians reported that producers generally did not test for the presence of diseases other than BVDV in PIs (combined median proportion of producers who test for diseases other than BVDV = 10%, 95% range 5–85%).

### 3.6. Welfare and PI Cattle

Most veterinarians (*n* = 19) thought that PI cattle had compromised welfare, but only a small number (*n* = 3) believed that producers were concerned about PI welfare. Veterinarians considered the cost of disease and production losses caused by active BVDV infections were producers’ main concerns. When asked to describe their concerns about PI cattle, veterinarians (*n* = 13) stated that PI cattle were clinically unhealthy and likely to have early and painful deaths. Due to increased susceptibility to pathogens, they are subject to other comorbidities and once clinically ill, they are unlikely to be treated. In particular, veterinarians also reported an apparent association between diseases such as pneumonia and infectious kerato-conjunctivitis in herds following exposure to PI cattle, thus reducing herd welfare overall.

Veterinarians (*n* = 12) were aware that producers sometimes use “vampire vaccination” (collect blood from PI cattle to administer to BVDV naïve cattle to stimulate immunity). Veterinarians suggested that the motivation for the use of “vampire vaccination” was a perceived improvement of exposure (the number exposed can be controlled and is guaranteed) and a reduced welfare risk to PI cattle (as PI cattle are not required to live for longer than required for the blood collection). Two veterinarians suggested that the drivers for this activity were economic and practical benefits; other options such as commercial vaccination, are too expensive and ineffective. One veterinarian stated that if commercial vaccination was of a “high quality with high levels of long-lasting immunity, it would reduce the desire of producers to create their own cost-effective control measures.” Some veterinarians (*n* = 3) expressed concern for this practice due to the risk of spreading other diseases due to inadequate hygiene and a lack of awareness of disease risk among producers.

When asked for additional comments, veterinarians indicated that the willingness of producers to implement control was believed to differ between beef and dairy producers, with dairy producers more likely to appreciate the benefits of eliminating BVDV compared with beef producers. Some veterinarians also believed that trading patterns and neighbouring farms make the prevention of exposure unfeasible, and the resulting exposures keep herd seroprevalence high.

Some veterinarians (*n* = 3) thought that producers’ knowledge of BVDV, its production impacts and control measures was poor. Awareness of comorbidities in PI cattle was also believed to be low.

Approximately half of the veterinarians (*n* = 21) suggested that with veterinarian involvement, the management of BVDV on properties could be improved, and some veterinarians said that producer knowledge also needed improvement.

Additionally, there was a general opinion by veterinarians that the current available vaccine in Australia is too expensive and not sufficiently effective, with one veterinarian declaring that until alternative options become available “it is not fair to take away other practices” such as the use of PIs.

## 4. Discussion

This study highlighted that veterinarians’ knowledge of herd-level BVDV prevalence—a key parameter for surveillance to determine whether control measures are effective—in their workplace region is limited. They appeared uncertain; in general, classifying their area as having a “low” or “high” herd-level prevalence, as well as estimating broad lower and upper limits around this parameter. This study further highlighted that recommendations by veterinarians for BVDV control do not always align with producers’ preferred strategies, especially regarding the use of PI calves for deliberate exposure. However, although veterinarians generally do not support the use of deliberate exposure for BVDV control, some are willing to work with producers who use it. It also highlighted veterinarians’ concern for the welfare of PI cattle, the use of a previously undocumented form of deliberate exposure which we term “vampire vaccination,” and the risk of disease transmission (other than BVDV) between PI cattle and non-infected cattle.

Most veterinarians in this study believed that PI cattle have compromised welfare because they are clinically unhealthy and suffer early deaths. This was consistent with findings from previous studies which reported significant welfare implications due to ill-thrift, mucosal disease and predisposition to secondary diseases [22,36,37]. In the current study, veterinarians perceived that producer concern about the welfare of PI calves was low. This was also consistent with a previous study which found a lack of producer interest in welfare concerns associated with BVDV [38]. Authors found that unpredictable disease consequences and perceived costs of disease control influenced producer motivation, and that producers were more interested in production than animal welfare [37]. The current study also highlighted another welfare concern regarding the administration of blood collected from PI cattle to administer to BVDV naïve cattle as part of vampire vaccination. Veterinarians who were aware of this practice raised concerns about the risk of spreading diseases other than BVDV when using the vampire vaccination. Veterinarians believed that the use of this practice was driven by producers’ perception that it was a cost-effective vaccination which provided life-long immunity. As such, vampire vaccination provided a cheaper alternative to the current commercial annual vaccination. Further investigation of vampire vaccination as part of BVDV control is required to determine incidence, the costs and benefits to producers, and the impact on the welfare of cattle exposed to this practice.

Veterinarians identified a range of enteric, respiratory, and systemic pathogen risks that could also be transmitted from PI to BVDV naïve cattle in both beef and dairy systems, as well as infectious kerato-conjunctivitis (pinkeye) and lice in beef cattle. Wide variation of within-herd prevalence of enteric and respiratory diseases in southern Australia (including the current study region) has been previously documented [38,39,40,41]. Therefore, the range of diseases and similar variation in veterinarians’ estimations of prevalence of enteric and respiratory pathogens in the current study were expected. Although there have been studies of the health status of PI cattle and the presence of concurrent diseases in BVDV infected animals, there has been minimal research into the impact of secondary disease transmission from PI to BVDV naïve cattle and the subsequent effect on within-herd prevalence of disease. While difficult to quantify, this is worth investigation to understand these additional influences of introduction of PI cattle into BVDV naïve herds. It is possible that this is an underestimated impact of BVDV on herd health. Additionally, further studies are needed to determine the prevalence of BVDV within different regions and climate zones in Australia. Current literature identifies varied prevalence across Australia, without determining prevalence within a single climate zone or discerning between beef and dairy industries. Multiple other studies have cited a range of seroprevalences across Australia between 13–100% [9,10,42]. These studies involved different herd sizes across different Australian states, with likely differing herd management strategies that potentially contributed to the variation in prevalence [9,10,42].

Veterinarians are a crucial part of biosecurity at farm level through improving producer knowledge [29,43]. However, in this study, veterinarians were uncertain about the proportion of herds that were BVDV positive in their regions. Consequently, they were also unsure about the proportion of producers who used BVDV control and prevention methods, although the median estimate was consistent with a previous southern Australian study in which 36% of cattle producers implemented BVDV control [11]. However, this proportion might be influenced by concurrent control measures for another disease that are beneficial in the control of BVDV. Internationally, surveys of producers and veterinarians have indicated that insufficient knowledge is the most common reason for the absence of control protocols for BVDV [44]. Recent studies also indicated that Australian producers were considered to have a low knowledge of BVDV [11,17]. As comprehensive knowledge of a disease is needed for disease control at a national level, increased herd-level surveillance and education is essential for Australian BVDV control.

Most veterinarians in the current study did not support the use of PI calves for deliberate exposure to induce immunity. Instead, veterinarians usually recommended the use of vaccination and biosecurity (often in combination). This aligns with international standards, which recommend prevention programs that include development of herd immunity to BVDV [3,18,20]. Overall, little is known about deliberate exposure in Australia in terms of its incidence and efficacy in different herd types and environments. There are potential shortfalls in achieving BVDV disease control using deliberate exposure [15,23,45,46], such as incorrect timing, length of exposure and failure to ensure all susceptible animals seroconvert [15,22,45]. A previous survey of southern Australian producers found that deliberate exposure was less commonly used than vaccination, and was also generally inadequately managed when used [11]. In contrast, veterinarians in the current study reported that deliberate exposure was used relatively more commonly than the commercially available vaccination, especially on beef properties. Overall, this study highlighted that deliberate exposure is occurring without the recommendation of veterinarians and might have become a more popular option for BVDV control than commercial vaccination. This emphasises the need to investigate and assess drivers for this practice if the Australian cattle industry were to consider national control of BVDV.

Selection bias could have influenced the results of this study due to the targeting of participants who were members of cattle veterinary groups; those who responded to the survey might have been more likely to have a particular interest in BVDV control. In addition, we also acknowledge that veterinarians’ knowledge of how clients employ measures to control BVDV might only reflect clients with close working relationships with their veterinarians, rather than producers who, for example, have small farms that require less veterinary input or only engage veterinary services for emergency reasons. Studies have shown the producers with good working relationships with veterinarians and prior knowledge are more likely to engage in discussion and follow advice [33,47,48]. Although sample size was small, the aim of this survey was to gain a range of responses from veterinarians, rather than a statistically significant consensus. For the purposes of BVDV control, it is important to appreciate and understand this range, because even a relatively small proportion of people with particular KAPs can influence control of a disease such as BVDV. The representation of a range of views, rather than majority opinions, are a valuable contribution to gaining an understanding of the challenges associated with BVDV control.

## 5. Conclusions

Veterinarians’ recommendations generally acknowledge and reflect their concerns about the welfare associated with BVDV infection, but do not necessarily match producers’ preferred strategies for BVDV control and management in their regions. To improve the control and management of BVDV, alignment of attitudes and practices is required. We suggest that this could be achieved by improving the knowledge of both veterinarians and producers about local BVDV prevalence to provide a baseline for surveillance and improve certainty about the impacts within their region. These include the direct economic impacts of BVDV control as well as indirect economic impacts and poor welfare in affected cattle. In particular, the drivers and impacts of the administration of blood from persistently infected cattle to BVDV naïve cattle that were documented in this study are worth further investigation. Finally, the differences in veterinarians recommended practices and those that they observe on farms likely represent differences in values between producers and veterinarians. A much greater understanding of how these values arise and their influence on BVDV control is needed.

## Figures and Tables

**Table 1:** Cattle pathogens suggested as having an increased prevalence in herds in which a PI is introduced, grouped by affected body system, reported by veterinarians in a survey of veterinarians’ knowledge, attitudes and practices associated with bovine viral diarrhoea virus management on farms in the temperate zone of south-east Australia in 2019.

Body System	Pathogen or Disease
Enteric	Coccidiosis
	Colibacillosis
	Cryptosporidiosis
	Neonatal Diarrhoea
	Salmonellosis Rotavirus
Respiratory	Bovine Respiratory Disease
	Mycoplasmosis Pneumonia
Systemic	BVDV
	Leptospirosis Septicaemia
	Theileriosis
Other	Lice
	Pinkeye (infectious bovine keratoconjunctivitis)

## Supplementary Materials

The following are available online at https://www.mdpi.com/2076-2615/10/9/1630/s1, Figure S1: Count of veterinarians working on beef or dairy properties and the number of properties seen each week in a survey of veterinarians’ knowledge, attitudes and practices associated with bovine viral diarrhoea virus management on farms in the temperate zone of south-east Australia in 2019. Figure S2: Counts of veterinarians in each age group in a survey of the knowledge, attitudes and practices associated with bovine viral diarrhoea virus management on farms in the temperate zone of south-east Australia in 2019. Figure S3: Barplot of the number of tests reported as being used by veterinarians to detect the presence of BVDV, in a survey of veterinarians’ knowledge, attitudes and practices associated with BVDV management on properties in the temperate climate zone of south-east Australia in 2019. VNT = Virus neutralisation test, AGID = Agar Gel Immunodiffusion Assay, VI = viral isolation, IH = immunohistochemistry, PCR = Polymerase chain reaction, ELISA = enzyme-linked immunosorbent assay. Figure S4: Number of veterinarians reporting deliberate exposure to different stock types, in a survey of veterinarians’ knowledge, attitudes and practices associated with BVDV management on farms in the temperate zone of south-east Australia in 2019. Figure S5: Boxplots of the estimated within herd prevalence of the grouped diseases, prevalence of the grouped diseased in BVDV persistently infected cattle (PI), and the proportion of producers testing for the grouped diseases on beef (left) and dairy (right) properties, in a survey of veterinarians’ knowledge, attitudes and practices associated with BVDV management on farms in the temperate zone of south-east Australia in 2019. Other = lice and infectious bovine keratoconjunctivitis.
