# Supplementary material for: Veterinarians’ Knowledge, Attitudes and Practices Associated with Bovine Viral Diarrhoea Virus Control and Prevention in South-East Australia
Source: Animals (Basel). 2020 Sep 11;10(9):1630. doi: 10.3390/ani10091630 (PMC7552315; doi:10.3390/ani10091630)
Supplement: Supplementary file 1 [file animals-10-01630-s001.pdf]

animals

MDPI

Article

Veterinarians' knowledge, attitudes and practices associated with Bovine Viral Diarrhoea Virus control and prevention in south-east Australia

Claire McMorrow, [1,2] Allan J. Gunn, [1,2] Shahid Khalfan, [1] Marta Hernandez-Jover [1,2] and Victoria J. Brookes [1,2,*]

[1] School of Animal and Veterinary Sciences, Charles Sturt University, Wagga Wagga, NSW 2650, Australia; cv.mcmorrow@gmail.com (C.M.); algunn@csu.edu.au (A.J.G.); shahid.khalfan@gmail.com (S.K.); mhernandez-jover@csu.edu.au (M.H.-J.)

[2] Graham Centre for Agricultural Innovation (NSW Department of Primary Industries and Charles Sturt University), Wagga Wagga, NSW 2650, Australia

* Correspondence: vbrookes@csu.edu.au

Supplementary Materials

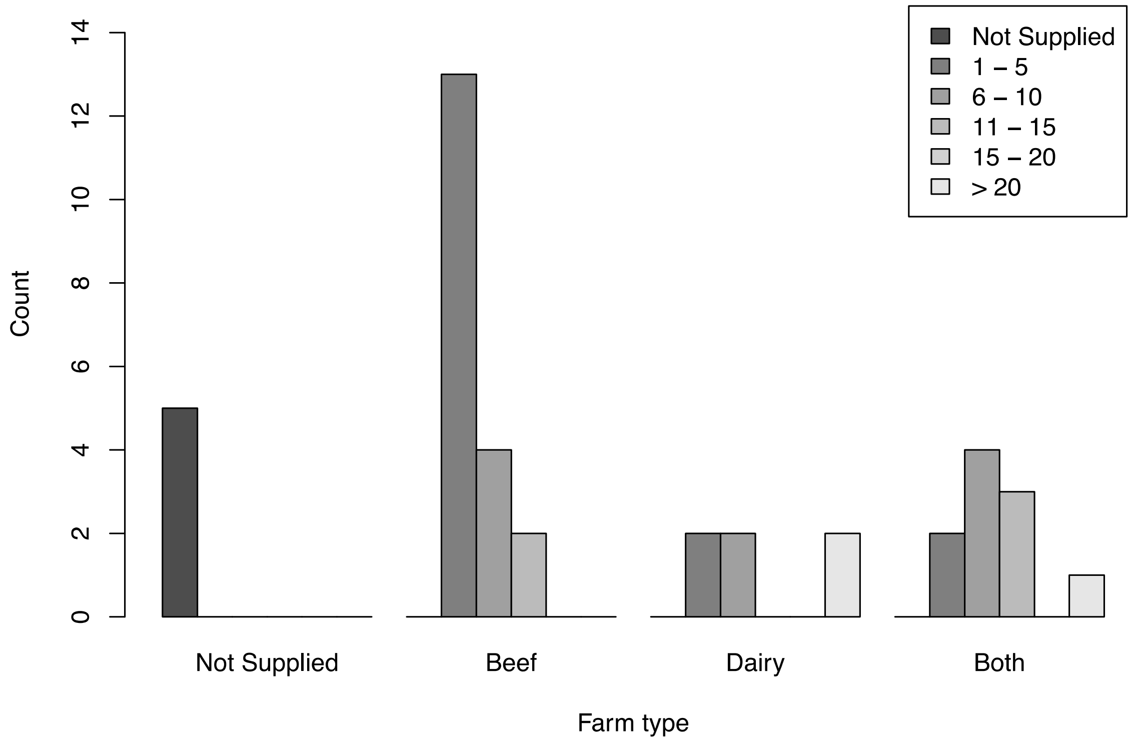

Figure S1: Count of veterinarians working on beef or dairy properties and the number of properties seen each week in a survey of veterinarians' knowledge, attitudes and practices associated with bovine viral diarrhoea virus management on farms in the temperate zone of south-east Australia in 2019.

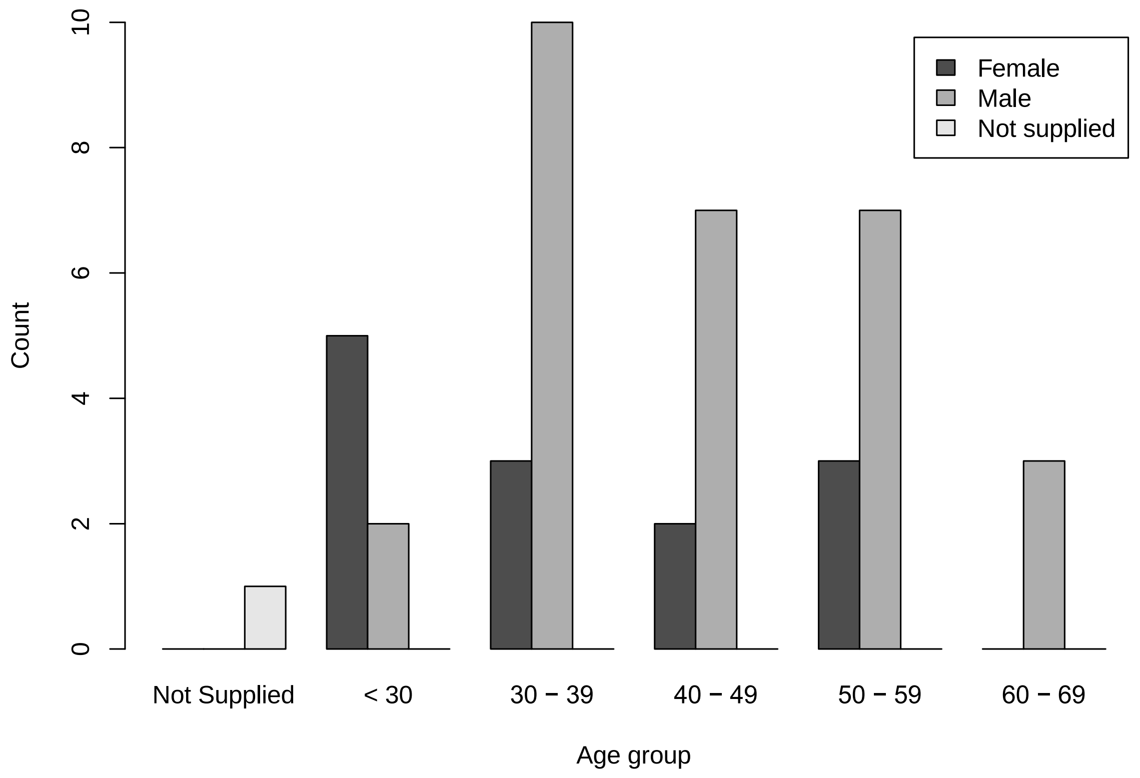

Figure S2: Counts of veterinarians in each age group in a survey of the knowledge, attitudes and practices associated with bovine viral diarrhoea virus management on farms in the temperate zone of south-east Australia in 2019.

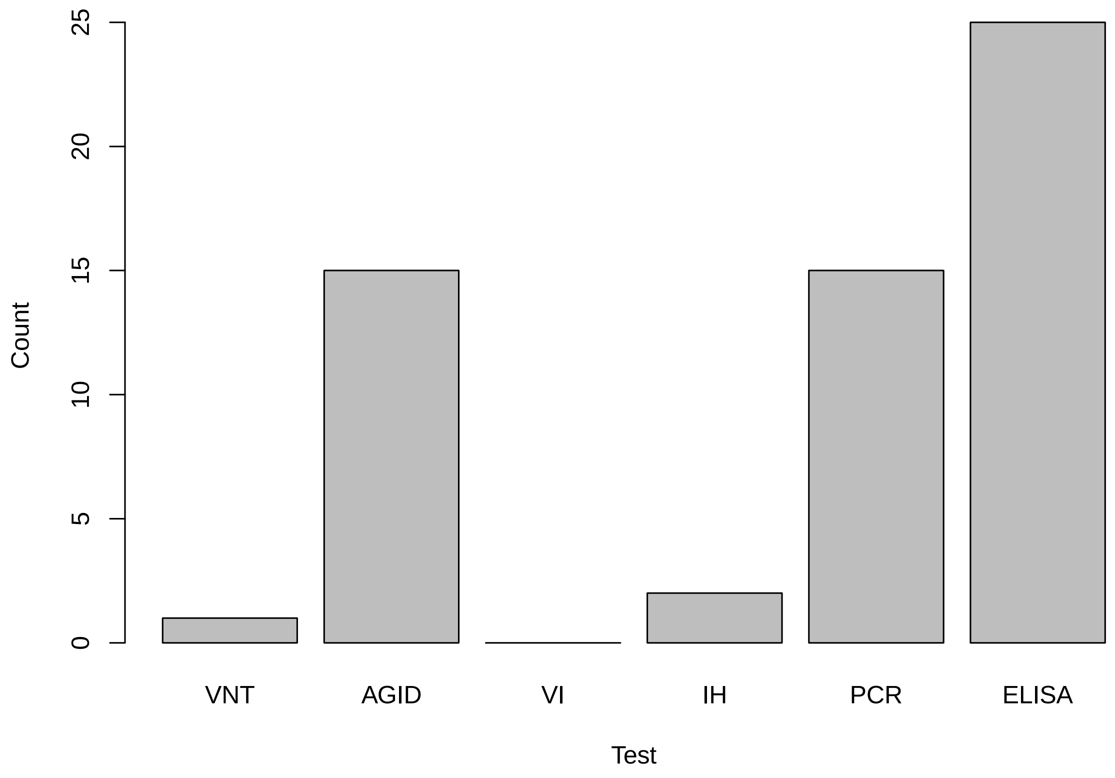

Figure S3: Barplot of the number of tests reported as being used by veterinarians to detect the presence of BVDV, in a survey of veterinarians' knowledge, attitudes and practices associated with BVDV management on properties in the temperate climate zone of south-east Australia in 2019. VNT = Virus neutralisation test, AGID = Agar Gel Immunodiffusion Assay, VI = viral isolation, IH = immunohistochemistry, PCR = Polymerase chain reaction, ELISA = enzyme-linked immunosorbent assay.

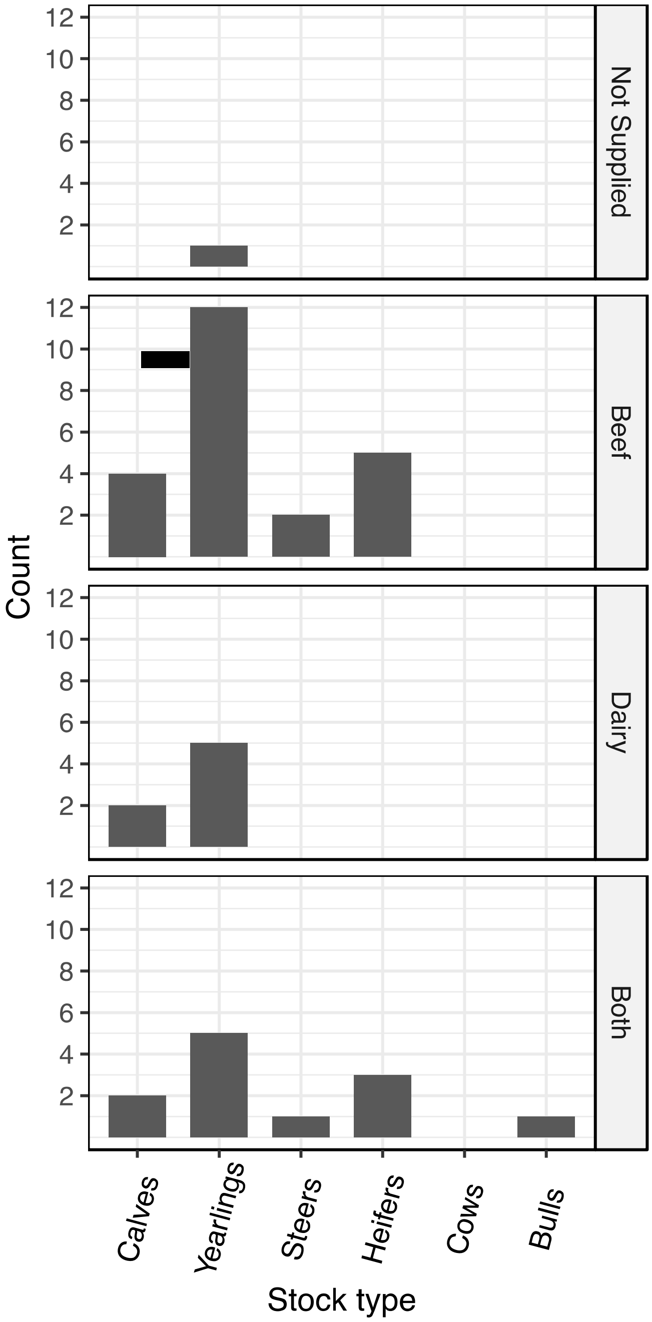

Figure S4: Number of veterinarians reporting deliberate exposure to different stock types, in a survey of the veterinarians' knowledge, attitudes and practices associated with BVDV management on farms in the temperate zone of south-east Australia in 2019.

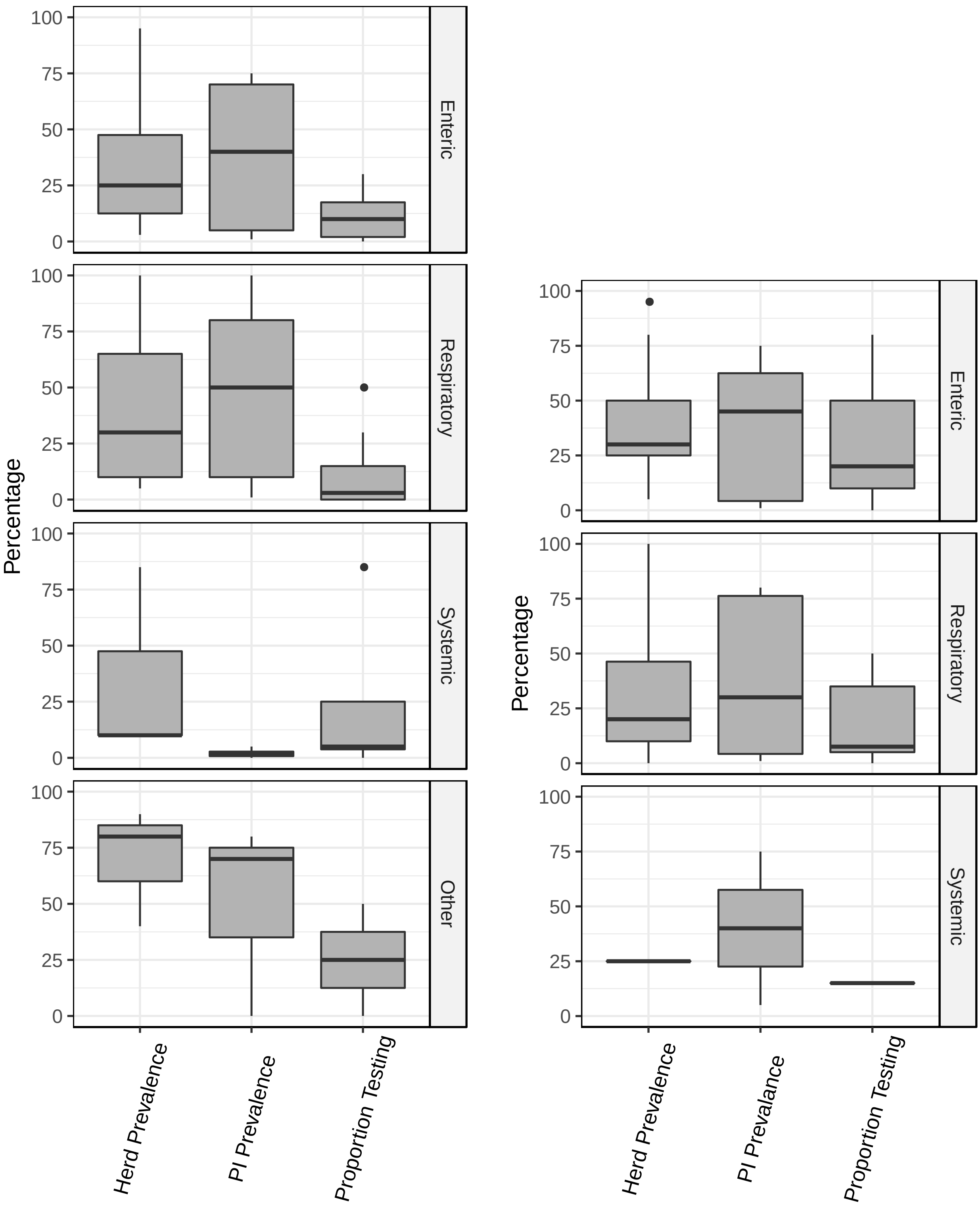

Figure S5: Boxplots of the estimated percentage within herd prevalence, BVDV persistently infected cattle (PI) prevalence and the proportion of producers testing for grouped diseases on beef (left) and dairy (right) properties, in a survey of the veterinarians' knowledge, attitudes and practices associated with BVDV management on farms in the temperate zone of south-east Australia in 2019. Other = lice and infectious bovine keratoconjunctivitis.
